# Investigation of Thermomechanical Properties of Hollow Glass Microballoon-Filled Composite Materials Developed by Additive Manufacturing with Machine Learning Validation

**DOI:** 10.3390/polym17111495

**Published:** 2025-05-28

**Authors:** Md Sakhawat Hossain, Sazid Noor Rabi, Sakib Mohammad, Kaden Cook, Farhan Chowdhury, Sabrina Nilufar

**Affiliations:** 1 School of Mechanical, Aerospace, and Materials Engineering, Southern Illinois University Carbondale, Carbondale, IL 62901, USA; mdsakhawat.hossain@siu.edu (M.S.H.); sazidnoor.rabi@siu.edu (S.N.R.); kaden.cook@siu.edu (K.C.); farhan.chowdhury@siu.edu (F.C.); 2 School of Electrical, Computer, and Biomedical Engineering, Southern Illinois University Carbondale, Carbondale, IL 62901, USA; sakib.mohammad@siu.edu

**Keywords:** hollow glass microballoon, stereolithography, representative volume element, finite element method, dynamic mechanical analysis, machine learning

## Abstract

Stereolithography (SLA) is a popular additive manufacturing (AM) method frequently used in research and various industrial sectors. The acrylate resin used in this research is renowned for its flexibility and durability, enabling the creation of flawless 3D-printed parts with exceptional mechanical properties. This study aims to enhance the thermomechanical properties of 3D-printed hollow glass microballoon (HGM)-filled composite materials by adding minimal HGM into the acrylate resin. We investigated the material properties through uniaxial compression tests, dynamic mechanical analysis (DMA), and scanning electron microscopy (SEM). To validate the results, a numerical investigation and a machine learning (ML) approach were carried out and compared with the experimental results. Adding a small number of microballoons increases compressive strength and stiffness. The viscoelastic behavior of the samples also provides an estimate of resilience at higher temperatures, considering the addition of filler material into the resin. Our study shows that the addition of 0.04% of HGM increased compressive strength by around 99.30% compared to the neat sample, while the stiffness increased by around 31.42% compared to the neat sample at 0.05% of HGM. It can also be estimated that the suitable range of HGM addition for the resin we used exists between 0.04% and 0.05%, where the materials achieve their maximum strength and stiffness. In addition, a predictive machine learning (ML) model, namely Random Forest Regressor (RFR), shows low mean squared error (MSE), mean absolute error (MAE), and excellent R^2^ scores, demonstrating the goodness of the model’s performance. This modern approach can guide us to selecting a suitable filler percentage for the photopolymer resin for 3D printing and making it applicable to different engineering prospects.

## 1. Introduction

Due to the prominent characteristics of composite materials, they have been widely used in aerospace, marine, automotive, building and construction, electrical and electronics, and various other sectors. One example of such a material that has extensive structural and functional applications is hollow glass microballoon (HGM)-filled epoxy composite materials [1]. These composite materials have low weight and density while having high strength [2]. These properties lead to the widespread usage of these materials in applications that need high impact endurance, resistance to prolonged hydrostatic pressure, buoyancy, and low water absorption. Manufacturing composite materials with HGM using a 3D printer provides several benefits compared to conventional methods. These benefits include enhanced design flexibility, streamlined fabrication, and reduced investment costs. Several studies have been carried out in this field using 3D printing. The 3D printing techniques applied most often for manufacturing these composites are Direct Ink Writing (DIW), Fused Filament Fabrication (FFF) or Fused Deposition Modeling (FDM), Directed Energy Deposition (DED), binder jetting, and Vat Photopolymerization (VPP) [3,4,5,6,7].

To create an object in FFF, the glass microballoon-filled precursor filament is first prepared, and later, the object is fabricated layer by layer. However, the multistep production process, high porosity, poor strength, and weak layer adhesion are the major drawbacks of adopting FFF to create syntactic foams [8,9,10]. Meanwhile, in Direct Ink Writing (DIW), thermoset samples exhibit greater strength and stiffness than thermoplastic samples. This method’s limitation is that the structures’ actual strength is less than the expected value. This may be attributed to void formation in the printing cartridge. The spherical shape of ceramic microballoon particles makes them suitable for binder jet printing due to their ideal spreading properties. However, for polymers, there are issues with compatibility with binders, causing problems in inter-layer adhesion, shrinkage, and warping, resulting in a poor surface finish [11]. According to ASTM, Vat Photopolymerization (VPP) is a 3D printing process [12] where UV light cures the liquid photopolymer resin, and the finished product is printed layer by layer onto the build platform [13]. Digital light processing (DLP), stereolithography (SLA), and two-photon polymerization (2PP) are the three types included in this VPP technique. This technique can overcome the shortcomings of the other 3D printing techniques. Its resolution and high accuracy, smooth surface finish, versatility of resin materials, speed and accuracy, capability to print complex geometries, post-processing optimization, good mechanical properties, and lower material wastage make it more popular than other 3D printing processes [14,15,16,17,18,19,20,21]. In a recent study, the DLP technique was used to develop and print a microballoon-filled resin to characterize thermomechanical properties [7]. They added a higher volume of microballoons to study the characteristics of the material and found that the distribution of fillers remains uniform with the variation of depth and print time. Moreover, they showed that the storage modulus is inversely proportional to the density of the printed material.

HGMs are utilized as a standard filler material in composite materials research for their low density and high stiffness [22]. Singh et al. used the FDM technique to create low-density and high-strength polymers with glass microballoons [23]. Their study found that the three-phase microstructures (matrix, microballoons, air voids) created due to the addition of HGMs have a more substantial impact on the strength of the composite, but not on the density. Another study used DIW technology to develop a biologically inspired core-shell (CS) architecture epoxy composite material constituted by a low-density core and stiff outer shell [24]. This unique composition enabled the use of the highest amount of HGMs, forming the lowest density core. Another group studied hollow microspherical particles by deploying the binder jetting technique. The mechanical and thermal properties of the 3D-printed parts were improved due to the viscous flow occurring during this sintering process [25]. A recent study successfully enhanced the mechanical and thermal properties of 3D-printed samples by utilizing DLP technology and mica-epoxy acrylate resin [26]. Their study focused on cost-effective printing for stronger and better materials. Here, one of our goals is to use the least amount of filler materials to improve the material properties.

Although a considerable number of studies have used various 3D printing methods, they have primarily focused on experimental analyses by manipulating resin properties with the addition of filler materials for better strength, lower density, and high thermal stability [27,28,29]. Only a few studies have developed numerical models to predict the behavior of resin materials based on filler type, volume composition, or void content [30,31]. These models can potentially revolutionize material design and characterization, enabling more efficient and innovative approaches for developing advanced polymeric resins for 3D printing.

ML has recently emerged as an excellent tool for detecting patterns in high-dimensional data analysis. It is now widely utilized in many fields, including AM, to make the process predictive and data-driven [32,33,34,35]. Implementing ML methods to better understand the effect of material constituents on material properties during 3D printing processes not only helps develop novel materials but also provides a new avenue for future studies for refining the existing AM processes. Accordingly, we have developed an ML model in this study that can readily predict the behavior of 3D-printed materials based on the amount of filler content.

In the present work, we aim to fabricate an HGM-filled composite material by leveraging the SLA technique with various HGM volume fractions and characterize its thermomechanical behavior. A water-washable photopolymer resin is used in this study, while K25 glass microballoon is considered the filler material. A uniaxial compression test was carried out with various volume fractions of the HGM to assess the material’s strength and stiffness. Also, for thermal analysis, dynamic mechanical analysis (DMA) was performed with different HGM volume fractions. A 3D model was generated using a representative volume element (RVE) [36,37,38], where the microballoons are placed randomly from 0.01% to 0.07%. An ML approach was employed to validate the experimental results and numerical simulations. Our findings, along with these FEA models and ML methods, contribute to a deeper understanding of the thermomechanical characteristics of these HGM-filled composites.

## 2. Materials and Methods

### 2.1. Materials

In this investigation, water-washable model grey resin (an acrylate-based photopolymer resin) from Phrozen technology of 405 nm wavelength (Hsinchu, Taiwan) was used as a matrix material. K25 glass microballoons (average diameter about 55 μm and density 0.25 g/cc) were used from 3M Scotchlite™ (St. Paul, MN, USA) as a filler material. Table 1 shows the composition of the samples.

### 2.2. Preparation of the Samples

The resin was prepared in a dark room, avoiding exposure to light. First, the Phrozen water-washable model grey resin was dispensed into a 50 mL aluminum foil-wrapped tube, and the glass microballoons were added to the resin as per the required amount. The microballoons were introduced slowly to avoid the risk of agglomeration, microballoon damage, and void formation [39,40,41,42,43]. Later, the resin-microballoon mixture was mixed in an ultrasonic mixer for 10 min at 30 °C. After that, the resin mixtures were put over the orbital shaker (Dubuque, IOWA, USA) for 30 min for further mixing. For the sample fabrication, the CAD models were created in Autodesk Inventor Professional 2024 (San Francisco, CA, USA). The compression samples were designed as per the ASTM D695-15 standard [44]. Later, the design file was sliced in Chitubox V2.0 (Chitubox, Shenzhen, China) slicer software for the final printing. The test specimens were printed using an LCD printer (Phrozen Sonic Mini, Phrozen 3D, Hsinchu City, Taiwan) of 405 nm wavelength, where the slice thickness of the samples was set to 100 µm, and the bottom exposure time was set to 30 s. The printer used a bottom-up printing method, and the compression samples were printed vertically. After printing, the samples were cleaned with isopropyl alcohol. They were post-cured for 30 min in the ANYCUBIC curing chamber of 405 nm wavelength (Shenzhen, China) for proper polymerization of the 3D-printed samples. Table 2 shows a summary of the printing parameters used in this study.

### 2.3. Mechanical Testing

We used a 30 kN MTS (Eden Prairie, MN, USA) Universal Testing Machine for the compression test of the samples, with a set strain rate of 1.27 mm/min. We tested at least five samples for our compression tests. The test continued until the samples were completely broken. The failure analysis of the samples was performed under applied load and stress concentration on the parts, which were further observed in scanning electron microscopy (Quanta FEG 450) (Waltham, MA, USA) coupled with energy-dispersive X-ray spectroscopy (EDS) (Concord, MA, USA) [45].

### 2.4. Dynamic Mechanical Analysis (DMA)

For the thermomechanical testing, dynamic mechanical analysis (DMA) was performed in DMA 850 from TA Instruments (New Castle, DE, USA), where a three-point bending mode was selected for the test. The temperature ramp mode was set to increase from 25 °C to 120 °C at a heating rate of 5 °C per minute to observe the thermomechanical properties of the samples, as used in our previous study [2]. Additionally, a 1 Hz frequency was used to apply periodic loading while maintaining an amplitude of 30 μm. The materials’ elastic and viscoelastic properties were obtained through the storage modulus (E′) and damping factor (tan δ) from the DMA results. The glass transition temperatures (T_g_) were also determined through this DMA test. For the DMA, sample dimensions were (56 × 12 × 2 mm^3^), following the ASTM D7028-07 standard [46], and we tested at least five samples.

### 2.5. Numerical Analysis

**Figure 1:**
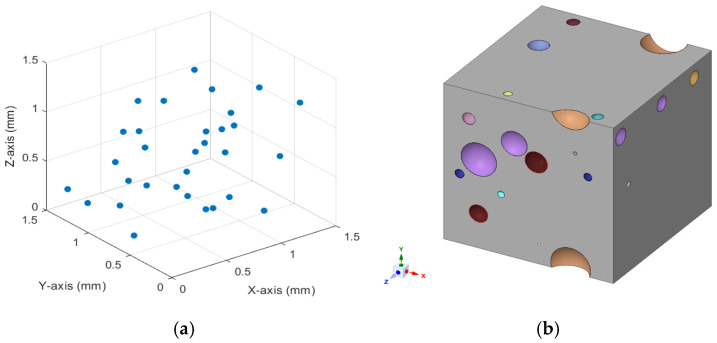
FEA model showing (**a**) distribution of microballoon in RVE at 0.04% and (**b**) distribution of microballoon in three-dimensional view.

For numerical analysis, MATLAB 2023 (Natick, MA, USA) was used to generate a location for each microballoon within a (1.5 × 1.5 × 1.5 mm^3^) model, which would serve as a representative volume element (RVE) for finite element analysis (FEA) shown in Figure 1a,b. The algorithm calculates the maximum number *n* of 55 μm diameter microballoons that would fit within the RVE at the specified volumetric particle concentration. A random coordinate was then generated for each *n* particle. An iterative loop was then employed to ensure that no two particles were overlapping at each random coordinate and that no particle overlapped with the RVE’s volumetric boundary. The Lubachevsky–Stillinger algorithm (LS) and Random Sequential Adsorption (RSA) are the two standard methods that can be used to distribute the microballoons randomly [43,47]. In our previous study [2], we used the LS algorithm; therefore, the LS algorithm has been used again here for the iterative loop. Once this iterative process generated a satisfactory set of coordinates, they were placed into a comma-separated value spreadsheet file for importing into FEM software.

**Figure 2:**
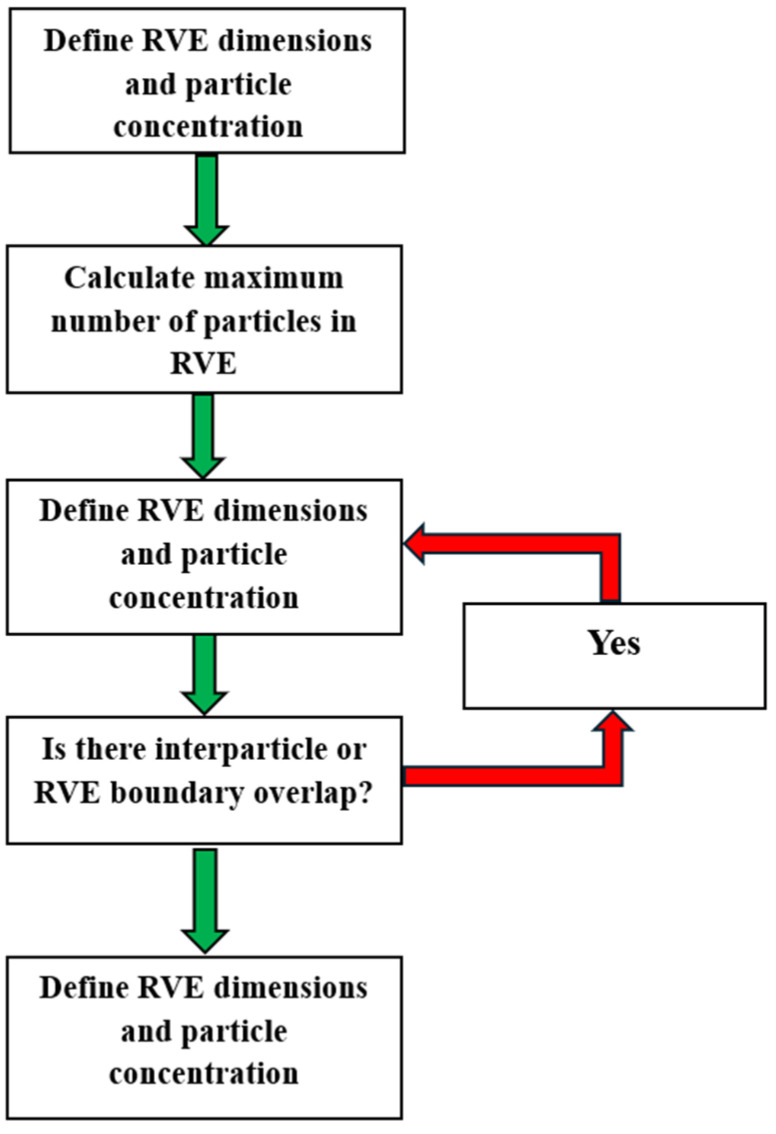
The flowchart for generating the random location of the glass microballoons in RVE using the LS algorithm.

Figure 2 provides a simple visualization of the generation algorithm used. For this model’s initial development and testing, the 0.04 vol.% particle inclusion was deliberately chosen, based on its demonstrated compressive strength compared to other concentrations. Using Autodesk Inventor 2024 (San Francisco, CA, USA), a VBA script was written to translate each generated coordinate into a 3D model of the set of hollow microballoons at their respective locations within the RVE. A particle diameter of 55 μm and a wall thickness of 0.731 μm were used for the spherical models. This coordinate model was then exported to Ansys SpaceClaim to be placed and meshed within a cube, effectively generating the desired RVE. SpaceClaim’s Material Designer function was also used to define and analyze the material as a parametric particle composite. The results obtained from this material analysis were used in subsequent evaluations.

### 2.6. Analysis by Machine Learning Model

To predict stress from strain values, we utilized an RFR model [48]. Using RFR, we predicted (1) stress–strain and (2) E′ and E″ properties for a range of temperatures using various microballoon volume fractions. We adopted an 80/20 training and test data split in both ML experiments and utilized MSE, MAE, and R^2^ scores to evaluate our models’ performance. Formulas for these three metrics are presented here:

(1) $$MSE=\frac{1}{n}\sum_{i=1}^{n}{\left(y_{i}-\dot{y_{i}}\right)}^{2}$$

(2) $$MAE=\frac{1}{n}\sum_{i=1}^{n}|y_{i}-\dot{y_{i}}|$$

(3) $$R^{2}=1-\frac{\sum_{i=1}^{n}{\left(y_{i}-\dot{y_{i}}\right)}^{2}}{\sum_{i=1}^{n}{\left(y_{i}-{\bar{y}}_{i}\right)}^{2}}$$

where:

n is the number of data points
$y_{i}$ is the actual value
$\dot{y_{i}}$ is the predicted value
${\bar{y}}_{i}$ is the mean of the actual values

Our code was developed in Python 3, and we utilized the NumPy [49], Pandas [50], Scikit-learn [51], and Matplotlib [52] libraries to formulate the study and visualize the results. Google Colab with a pro tier subscription was employed for the experiments.

## 3. Results and Discussion

### 3.1. Microstructural Characterization

**Figure 3:**
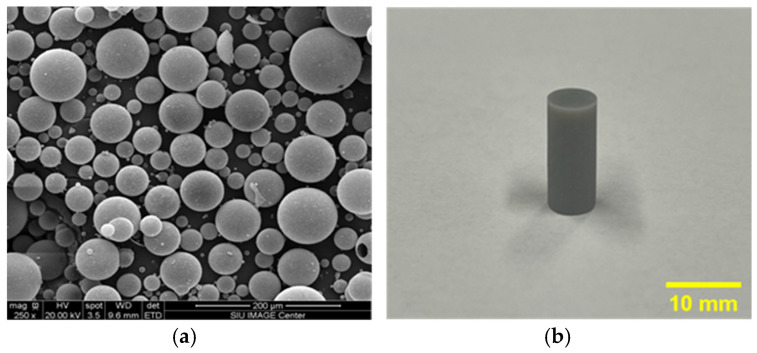
(**a**) Microstructure of K25 glass microballoons visible in SEM pictures at 250× magnification, (**b**) compression sample after 3D printing.

Figure 3a displays scanning electron microscopic (SEM) images of K25 glass microballoons, with magnifications of 250×, that provide information about the K25 glass microballoons. It is also shown in Figure 3a that, after SEM, the average size of glass microballoons is around 55–60 μm. Figure 3b displays the compression sample (6.35 mm × 12.70 mm) after the 3D printing.

### 3.2. Mechanical Properties

**Figure 4:**
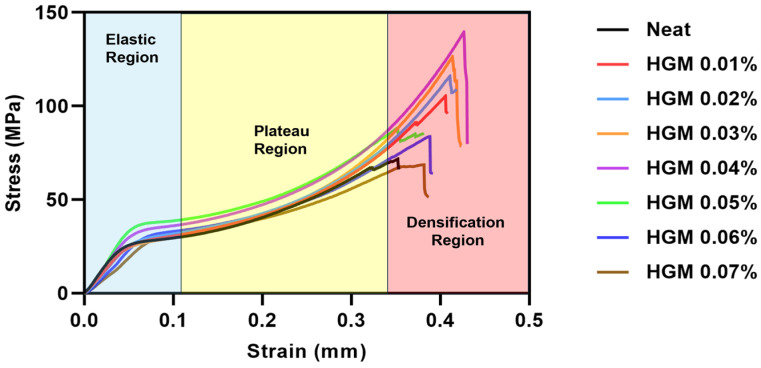
Representative experimental stress–strain curves from the compression testing of HGM composites with different volume fractions of microballoons. Blue, yellow, and red refer to the elastic, plateau, and densification regions, respectively.

We used HGMs from 0.01% to 0.07% by volume with Phrozen photopolymer and a neat sample containing no HGM for manufacturing these composites. Figure 4 illustrates the standard stress–strain curve at different HGM volume percentages. Three zones can be distinguished from these curves: the elastic, plateau, and densification regions [43]. The elastic deformation of the curve is represented by an area that is initially linear. This phenomenon occurs before the highest stress value and is considered the elastic region. After the elastic region, the plateau region appears where the internal structure of the materials begins to collapse, and a relatively flat stress–strain curve is obtained. Here, we can observe that the material is compressed for a long time in this region with a relatively small increase in stress. As a result, the materials absorb a high amount of energy in this region. The densification region appears just after the plateau region, where it shows higher strains. In this region, the highest peak of the curve is obtained, and the material’s behavior resembles a solid. We can also observe a sharp increase in the curve in this region, and the materials become much harder to compress [53].

**Figure 5:**
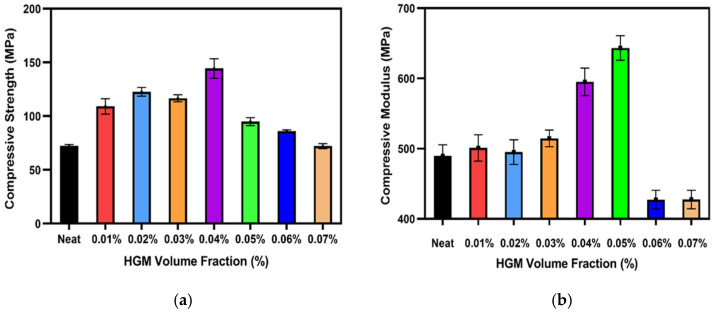
Effect of the microballoon volume fraction on (**a**) compressive strength; (**b**) compressive modulus.

In the plateau region, the microballoon starts to break, and the voids created due to the break are filled by the resin matrix. As a result, the materials tend to yield. We know that the volume percentage and density of the glass microballoons provide the matrix sufficient support, and their rupture causes matrix failure. This is indicated in the plateau region of the curves [2]. This plateau area also represents the amount of energy that the composite material can absorb. As a result, the material’s energy absorption can be boosted by employing microballoons with denser walls and larger densities [54]. Finally, the material reaches the densification region, and at this point a significant increase occurs in the stress, and the matrix turns into a bulk material. When HGMs are added, the material’s compressive strength and stiffness rise because of better interfacial adhesion with the matrix. Since it affects their total compressive strength and stiffness, the interfacial strength between the HGM and the matrix is crucial [22]. It is evident from Figure 4 that at 0.04%, the material reaches the highest compressive strength. However, when more fillers are added, compressive strength decreases gradually. A similar trend can be observed in Figure 5, where the maximum compressive modulus is obtained at 0.05% HGM but then decreases abruptly.

The reason could be attributed to the addition of higher volume percentages of HGM into the resin mixture, which makes the resin mixture more slurry-like. It might be due to the aggregation of filler materials, poor dispersion of filler materials, or higher viscosity of the resin–filler mixture that prevents the UV curing [39,55,56,57]. As a result, the interfacial bonding between the filler materials and the resin becomes weak and reduces the compressive strength and modulus [22]. The higher glass microballoon content also increased embrittlement, reducing the strain-to-failure and causing more pronounced structural collapse under load [58]. It can be inferred that between 0.04% and 0.05%, there is likely a filler material concentration where both the maximum compressive modulus and the compressive strength can be obtained. This indicates that there is an ideal range of HGM content for the proper polymerization that makes the sample both stronger and stiffer.

### 3.3. Failure Mechanism

**Figure 6:**
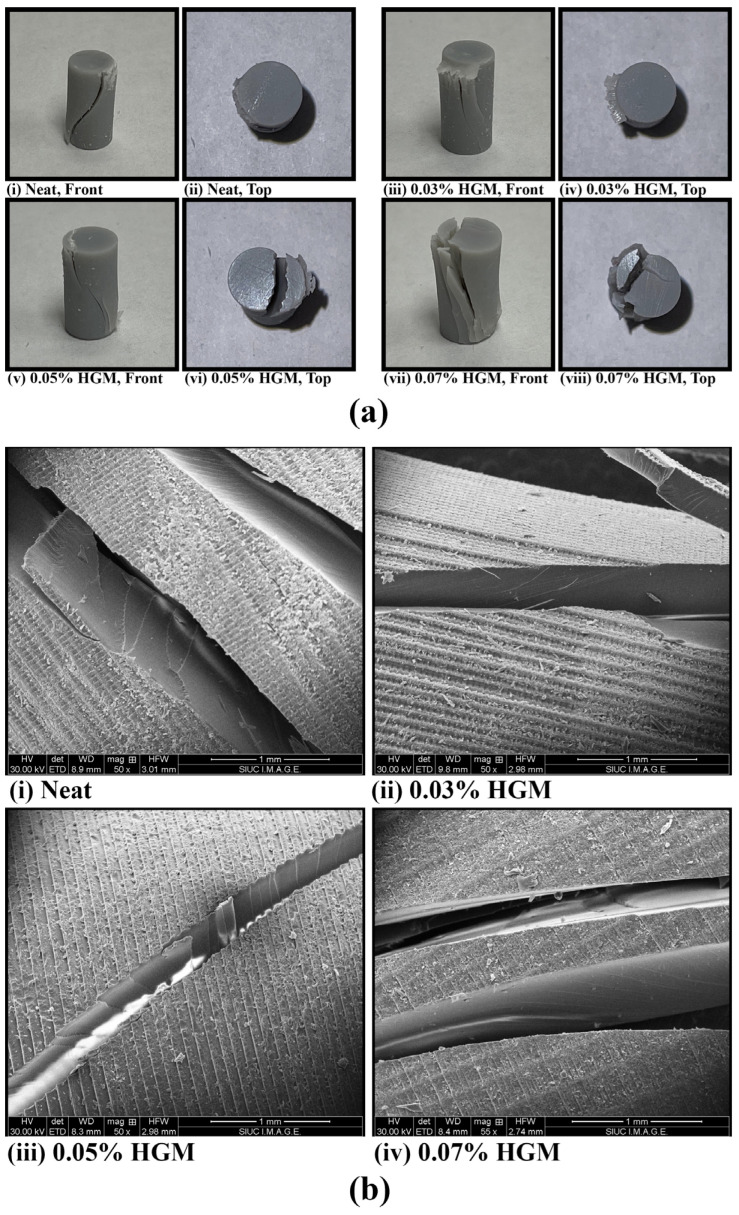
(**a**) Cracked sample images after compression test; (**b**) SEM images of failed samples.

The stress in the glass microballoon is significantly higher than that in the resin matrix. The primary causes of failure are the rupture of the microballoons and the development of microcracks in the matrix. Thus, the HGMs are the key constituents that bear most of the applied loads. When the samples are subjected to additional load, these microcracks and microballoon fractures grow, combine to produce a macrocrack, and eventually cause the samples to fail. Additionally, a macrocrack may form from the voids connecting neighboring microcracks in the matrix, as shown in Figure 6a. We have further shown the crack patterns for neat, 0.03% HGM, 0.05% HGM, and 0.07% HGM samples. Although there is no prominent difference among these samples in terms of failure mechanism, it is evident from Figure 6a that the 0.07% HGM samples have a more pronounced crack pattern, which is supported by the stress–strain curve in Figure 5. This is further supported by the SEM micrograph in Figure 6b, where the crack that largely extends through the matrix adjoins all the smaller cracks and forms a diagonal macrocrack.

The failed samples in Figure 6a show that a longitudinal cracking path is predominant, regardless of the amount of microballoons present. This indicates that the proportion of microballoon volume has no appreciable effect on how the object fails. Also, adding HGM created a few imperfections, leading to unwanted porosity and weak interfacial surfaces between the HGM and the matrix [22]. It resulted in crack propagation inside the sample, shown in the SEM images in Figure 6b. The bond between the HGM and the matrix should have good adhesion to enable the crack to propagate along the matrix or possibly cause an HGM fracture. The presence of longitudinal cracks indicates that cracks propagate within the matrix [43]. It has been found that the compressive modulus increases with a higher microballoon volume fraction, peaking at 0.05% before decreasing. It is evident that glass microballoons are extensively dispersed in the resin at low-volume fractions. As stress concentrates in the matrix around the top and bottom of microballoons, the microcracks tend to spread toward these locations and generate a longitudinal macrocrack.

### 3.4. Dynamic Mechanical Analysis Results

**Figure 7:**
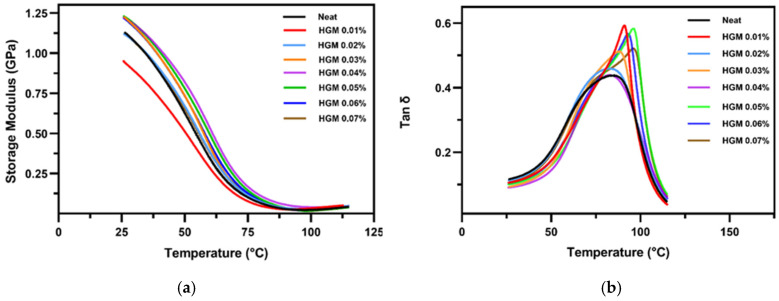
DMA results of HGM-filled composites at several volume fractions showing (**a**) storage modulus; (**b**) damping factor (tan δ).

The viscoelastic behavior of materials changes with temperature [59]. The DMA results express the structures and viscoelastic behavior of the materials to determine their damping characteristics and relative stiffness [60]. The interfacial bonding and thermal relaxation of the polymers and composites with temperature, strain rate, stress, dynamic/complex viscosity, and creep compliance are also expressed by DMA [61]. Overall, dynamic mechanical properties also alter significantly with minor changes in the physical qualities of the materials. More research has been conducted on how temperature and filler composition affect the storage modulus and damping qualities of 3D-printed samples. Figure 7a shows the storage modulus with several volume fractions along with the neat samples. It shows that, with increasing temperature, the storage modulus decreases, and a sharp decrease zone is around the glass temperature region. Above 90 °C, the curves approach the flow zone where the storage modulus stabilizes to a very low value with very little change. It is observed that the storage modulus becomes higher compared to the neat sample before the glass transition temperature; however, this trend is not uniform due to the anisotropic nature of the 3D-printed composite materials. There are several reasons behind this anisotropic nature. It can happen due to improper curing and post-processing, loss of interfacial bonding, non-homogeneous filler material distribution in the resin, and so on [55,62,63]. However, after the glass transition temperature region enters the rubbery state, the trend reverses, and the lower volume fraction shows higher values. This is due to the softening of the matrix. Adding HGM initially causes the matrix to be more brittle, which explains the variance in storage modulus. However, the storage modulus increases when HGM is added at concentrations over 0.01%. The improved low temperature of the composites is attributed to the restricted movement of polymer chains due to the interaction between the photopolymer resin and microsphere particles [25]. Thus, it is evident that introducing a small amount of HGM reinforces the matrix.

Figure 7b shows the tan δ variation, the ratio of storage and loss modulus as a function of temperature. It shows that, as the microballoon volume percentage increases, the glass transition temperature (T_g_) shifts to the upper temperature region. However, this trend is different for 0.04% HGM. It is also evident in Figure 7b and Table 3, respectively, that the damping coefficient and glass transition temperature are lower when 0.04% of HGM is added into the resin, while at 0.05%, both these properties are much higher. However, for HGM 0.04%, the curve is more spread out, indicating better damping characteristics. Recent studies have found that using a high-volume percentage of HGMs can decrease the value of tan δ compared with the neat sample [64]. In contrast, our study has found that a small amount of HGM addition can enhance damping behavior for a shorter temperature range compared to neat resin. This can be due to the aromatic nature of the molecular chain, which improves the energy dissipation in the resin [65]. Microballoon addition usually causes increased glass transition temperature and higher damping behavior. However, there is a decrease for the 0.02% and the 0.04% cases. This can happen due to improper polymerization of the resin. Moreover, plasticization might occur when spherical fillers collapse into clustered particles [43]. However, due to the anisotropic nature of the 3D-printed composite materials, the increase in the damping factor is not continuous.

When HGM concentrations are low, microballoons limit polymer chain movement, which improves thermal stability and elevates the glass transition temperature (Tg). The intermediate concentration of HGMs leads to agglomeration or incomplete dispersion, which creates heterogeneities that disrupt the polymer matrix and reduce network formation efficiency, resulting in a slight decrease in Tg. The glass transition temperature (Tg) rises as well-dispersed microballoons create a physical barrier and expanded interfacial area, which limits polymer chain movement at elevated HGM concentrations. Therefore, the Tg variation demonstrates a balance between reinforcement-induced restriction and disruption of matrix continuity, rather than a linear correlation with filler content.

### 3.5. Numerical Simulation with ANSYS

**Figure 8:**
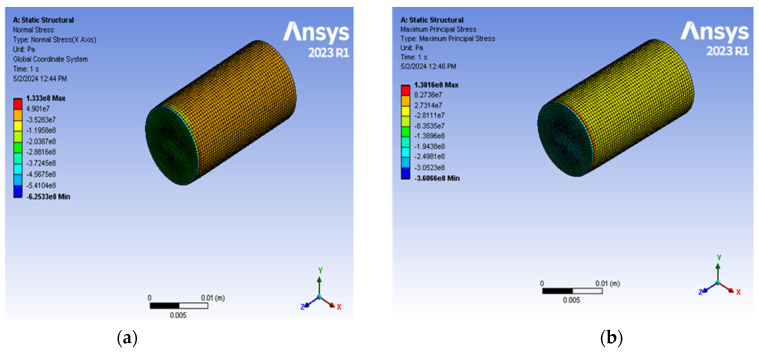
(**a**) Normal stress gradient within cylindrical compression sample; (**b**) maximum principal stress gradient within.

In our numerical simulation, the RVE model was generated by the software package ANSYS 2023 R1, and the viscoelastic properties of the material collected from our experimental results were added as Prony series parameters. We generated a sample drawing using the ASTM D695-15 standard [44]. All the required analyses for meshing pre-processing, solution, and after-meshing were performed [66]. Here, a tetrahedral mesh was used for the analysis with 2,43,216 nodes and 58,599 elements. The RVE model of the 3D-printed material was subjected to symmetrical boundary conditions. The bottom surface was fixed while the top surface was subjected to a predefined load equivalent to a strain rate comparable to our experiments. After meshing the model and adding the appropriate amount of force axially on one side, we obtained the stresses generated at the fixed interface of the sample. The results are depicted in Figure 8a,b. As shown in Figure 8a, the normal stress of the sample with HGM 0.04% is 133.3 MPa. In contrast, the experimental data indicate a strength of 139.03 MPa, with an error percentage of 4.30%. Compressive modulus is another essential property of a material’s ability to resist deformation when subjected to compressive forces. We found, using numerical analysis, that the compressive modulus for HGM 0.04% is 595.23 MPa, while the experimental value is 585.18 MPa (the error percentage is 1.72%), with Poisson’s ratio ν = 0.35. So, comparing the result with the experimental data, we can verify that our simulation values are aligned and are only offset by a negligible margin. This phenomenon indicates that our experiments are consistent and can be validated with a model that simulates the ideal conditions created by the material we have used.

Although our model is closely aligned with the experimental results, some limitations exist. We have assumed that there is no polydispersity, meaning that all the molecules or particle sizes are approximately of uniform size, which might not be the case, but is a close approximation for our samples. Moreover, we ignore the presence of voids as the amount of microballoons is small, making it negligible for such mixtures [47].

### 3.6. Prediction with ML Model

**Figure 9:**
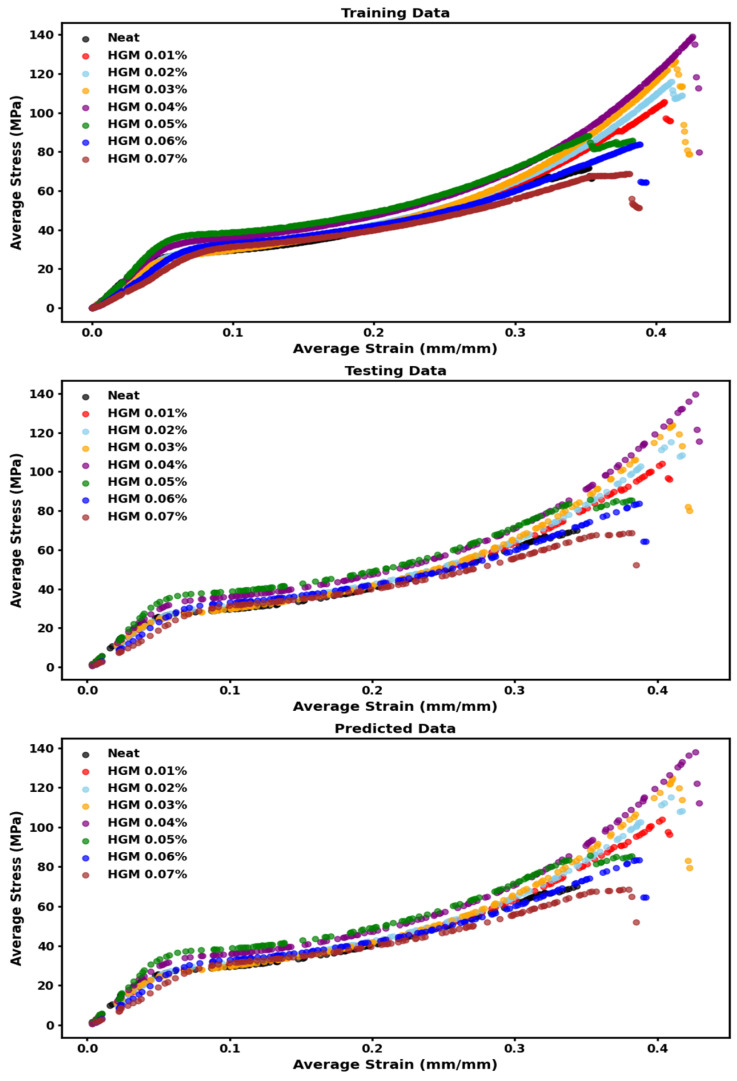
Training, testing, and predicted data points for stress vs. strain.

In this section, we present the prediction results using our RFR models for various microballoon concentrations. First, we will show the results for predicting stress from the strain values, followed by predicting E′ and E″ at different temperatures. In Table 4, we show that our model had excellent goodness of fit for predicting the stress from strain values with minimal error values (as evident from MSE and MAE values). In Figure 9, we show that the predicted data closely follow the test data.

**Figure 10:**
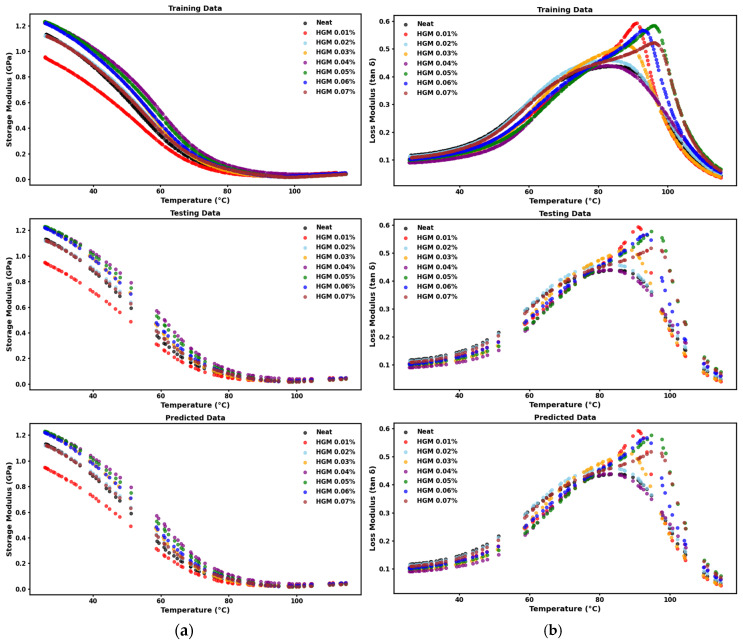
Training, testing, and predicted data of (**a**) storage modulus and (**b**) tan δ values at different temperatures.

Next, we predicted E′ and tan δ at different temperatures. From Table 5 and Figure 10, we observed that MSE and MAE values were negligible and displayed almost perfect R^2^ values for E′ and tan δ. This indicates that the regressor could account for the variability in the training dataset and performed very well for unseen E′ and tan δ values at different temperatures.

The similarity in the trends between the testing and predicted data in Figure 9 and Figure 10 is expected, as the predicted curves are generated using the same input conditions as the test set. The testing data consist of unseen instances from all concentrations, and the predicted outputs mirror the model’s response to those input data points. The strong overlap between the two confirms that the model successfully learned the underlying patterns and generalizes well within the given composition range. This extent of agreement highlights the efficacy of our model in interpolating stress–strain and modulus-temperature behavior across various HGM concentrations.

## 4. Conclusions

In this study, the thermomechanical behavior of a 3D-printed composite material was studied. This study shows that HGM significantly impacts both the mechanical and thermal properties of the composites. The results show that at 0.04% HGM loading, its compressive strength increases around 99.30% compared to the neat sample, while the stiffness increases around 31.42% at 0.05% of HGM compared to the neat sample. Additionally, the DMA analysis reveals that as the HGM increases, the storage modulus also increases, and at 0.05%, the glass transition temperature is significantly higher compared to the neat sample. The experimental results also show that at 0.04%, the compressive modulus is 595.23 MPa, while the simulation results show 585.18 MPa, and the error percentage is 1.72% of the experimental result. An ML approach has also been used to predict stress–strain curves with E′ and tan δ at different temperatures. The models’ performance was evaluated with MSE, MAE, and R^2^ scores, which show excellent performance in predicting the output variables. However, an extensive study with numerical analysis is required, where both the compressive strength and compressive modulus are studied with all HGM volume fractions and compared with the experimental results. An advanced DMA simulation should be performed to compare the viscoelastic behavior of the material with the experimental result. In addition, by changing the composition of the resin and the amount of HGM, a comprehensive relationship between microballoon volume fraction and thermomechanical behavior can be established.

## Figures and Tables

**Table 1:** Composition of samples prepared for study.

Samples	Composition
Neat	Resin without any glass microballoon
HGM 0.01%	0.01 vol% glass microballoon with neat resin
HGM 0.02%	0.02 vol% glass microballoon with neat resin
HGM 0.03%	0.03 vol% glass microballoon with neat resin
HGM 0.04%	0.04 vol% glass microballoon with neat resin
HGM 0.05%	0.05 vol% glass microballoon with neat resin
HGM 0.06%	0.06 vol% glass microballoon with neat resin
HGM 0.07%	0.07 vol% glass microballoon with neat resin

**Table 2:** Printing parameters of samples.

Parameter	Value
Slice thickness	100 µm
Number of base layers	5
Bottom exposure time	30 s
Curing time of the layers	10 s
Lifting distance	6 mm
Lifting speed	60 mm/min
Retraction speed	150 mm/min

**Table 3:** Comparison of storage modulus E′, transition temperature Tg, and loss modulus E″.

Samples	E′ at 30 °C (GPa)	E′ at 105 °C (GPa)	T_g_	E″ at 105 °C (GPa)
Neat	1.074	0.028	85	0.004
HGM 0.01%	0.887	0.041	91	0.005
HGM 0.02%	1.070	0.036	82	0.005
HGM 0.03%	1.169	0.034	88	0.004
HGM 0.04%	1.179	0.041	82	0.007
HGM 0.05%	1.180	0.026	96	0.006
HGM 0.06%	1.162	0.033	93	0.006
HGM 0.07%	1.067	0.024	96	0.005

**Table 4:** Evaluation metrics for predicting stress–strain values for different micro-balloon concentrations.

Samples	MSE	MAE	R^2^ Score
Neat	0.0212	0.0858	0.9999
HGM 0.01%	0.0237	0.1083	1.0000
HGM 0.02%	0.0185	0.0999	1.0000
HGM 0.03%	0.0430	0.1324	0.9999
HGM 0.04%	0.1259	0.1452	0.9999
HGM 0.05%	0.0281	0.0925	0.9999
HGM 0.06%	0.0170	0.0834	1.0000
HGM 0.07%	0.1282	0.1056	0.9996

**Table 5:** Evaluation metrics for predicting Storage and tan δ at different temperatures.

	MSE		MAE		R^2^ Score	
Samples	Storage Modulus	tan δ	Storage Modulus	tan δ	Storage Modulus	tan δ
Neat	0.0000	0.0000	0.0017	0.00	0.9999	0.9997
HGM 0.01%	0.0000	0.0000	0.0012	0.0015	0.9999	0.9993
HGM 0.02%	0.0000	0.0000	0.0014	0.0010	0.9999	0.9997
HGM 0.03%	0.0000	0.0000	0.0016	0.0012	0.9999	0.9997
HGM 0.04%	0.0000	0.0000	0.0015	0.0010	0.9999	0.9997
HGM 0.05%	0.0000	0.0000	0.0016	0.0017	0.9999	0.9994
HGM 0.06%	0.0000	0.0000	0.0015	0.0012	0.9999	0.9996
HGM 0.07%	0.0000	0.0000	0.0012	0.0011	0.9999	0.9995

## Data Availability

Data and results of the ML studies can be found at https://github.com/sakibmohammad/Polymer-HGM (accessed on 25 May 2025).
